# Enhanced thermoelectric performance of β-Zn_4_Sb_3_ based nanocomposites through combined effects of density of states resonance and carrier energy filtering

**DOI:** 10.1038/srep17803

**Published:** 2015-12-15

**Authors:** Tianhua Zou, Xiaoying Qin, Yongsheng Zhang, Xiaoguang Li, Zhi Zeng, Di Li, Jian Zhang, Hongxing Xin, Wenjie Xie, Anke Weidenkaff

**Affiliations:** 1 Key laboratory of Materials Physics, Institute of Solid State Physics, Chinese Academy of Sciences, 230031 Hefei, PR~China; 2 Institute of Materials Science, University of Stuttgart, 70569 Stuttgart, Germany; 3 University of Science and Technology of China, 230026 Hefei, PR~China; 4 Hefei National Laboratory for Physical Sciences at Microscale, Department of Physics, University of Science and Technology of China, Hefei 230026, P.R. China; 5 Collaborative Innovation Center of Advanced Microstructures, Nanjing University, Nanjing 210093, PR~China

## Abstract

It is a major challenge to elevate the thermoelectric figure of merit ZT of materials through enhancing their power factor (PF) and reducing the thermal conductivity at the same time. Experience has shown that engineering of the electronic density of states (eDOS) and the energy filtering mechanism (EFM) are two different effective approaches to improve the PF. However, the successful combination of these two methods is elusive. Here we show that the PF of β-Zn_4_Sb_3_ can greatly benefit from both effects. Simultaneous resonant distortion in eDOS via Pb-doping and energy filtering via introduction of interface potentials result in a ~40% increase of PF and an approximately twofold reduction of the lattice thermal conductivity due to interface scattering. Accordingly, the ZT of β-Pb_0.02_Zn_3.98_Sb_3_ with 3 vol.% of Cu_3_SbSe_4_ nanoinclusions reaches a value of 1.4 at 648 K. The combination of eDOS engineering and EFM would potentially facilitate the development of high-performance thermoelectric materials.

Thermoelectric materials play an important role in cooling of electric devices and power generation from waste heat^1^,^2^,^3^,^4^,^5^,^6^,^7^. The conversion efficiency of a thermoelectric material is characterized by the figure of merit, ZT, defined as: ZT = (S^2^/ρκ)T, where S, ρ, κ are the thermopower, electrical resistivity, and thermal conductivity, respectively. Typically, there are two ways to improve the ZT of thermoelectric materials: one is to lower the thermal conductivity κ^8^,^9^,^10^,^11^,^12^ and the other is to boost the power factor PF = S^2^/ρ^13^,^14^,^15^,^16^. While a variety of techniques is available to reduce the thermal conductivity, such as embedding nanostructures in bulk materials^10^,^11^, the fundamental challenge is to boost PF, for instance by increasing the thermopower S. According to the Mott equation, the thermopower S of a degenerate semiconductor can be expressed as:


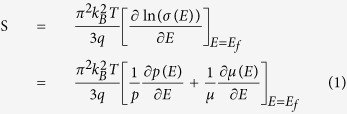


with the carrier mobility μ(E) = qτ/m_d_*, where σ is the electrical conductivity, q the carrier charge, E the energy, p(E) and μ(E) energy dependent carrier density and mobility, λ the scattering parameter, k_B_ the Boltzmann constant, m_d_* the effective mass and E_f_ the Fermi energy. With the approximation of a free-electron gas and assuming an exponential dependence of the scattering parameter λ on the relaxation time τ, i.e. τ = τ_0_E^λ−1/2^ (here τ_0_ is an energy-independent constant), Eq.(1) can be written as:


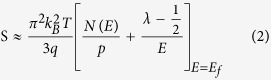


where N(E) is the electronic density of states (eDOS). Eq(2) implies that at a given carrier concentration, S can be enhanced by either increasing N(E), i.e. the eDOS at the Fermi level, or the scattering parameter λ, which corresponds to the energy filtering mechanism (EFM)^14^. Heremans *et al.* showed that after Tl-doping of PbTe, its ZT is doubled due to the enhancement of the thermopower^17^. This is attributed to the resonant distortion of the eDOS. Accordingly, the recently observed enhanced thermopower of Pr and Sm doped β-Zn_4_Sb_3_ can also be ascribed to the eDOS distortion of the host^18^,^19^.

On the other hand, Heremans *et al.* also observed an enhanced thermopower in PbTe-based nanocomposites containing Pb and Ag nanoparticles. This can be referred to the EFM^20^,^21^ revealed by the increase of the scattering parameter λ. Only recently, Zou *et al*. experimentally proved that the introduction of Cu_3_SbSe_4_ nanoinclusions increases the thermopower S of β-Zn_4_Sb_3_ by EFM^22^. Theoretical studies indicate that the resulting interface potentials of semiconductor-based nanocomposites with semiconducting^23^ or metallic^24^ nanoinclusions stimulate the EFM. Although both resonant eDOS distortion and EFM have been used separately to enhance the thermopower S of a specific material, a successful application of both effects at the same time has not been reported so far.

In this study, we show that the two approaches can be combined to improve the thermoelectric performance of β-Zn_4_Sb_3_. As it is known, β-Zn_4_Sb_3_ is one of the most promising thermoelectric materials on account of its low glasslike thermal conductivity and good electrical properties at moderate temperatures^25^,^26^,^27^,^28^,^29^; Cu_3_SbSe_4_ is another important thermoelectric material with a narrow band gap^9^,^30^,^31^,^32^. In order to induce resonant distortion of the eDOS, we substituted Pb for Zn in β-Zn_4_Sb_3_ forming β-(Zn_1-x_Pb_x_)_4_Sb_3_ (x = 0, 0.01, 0.02, and 0.03). On the other hand, we synthesized β-(Zn_1-x_Pb_x_)_4_Sb_3_-based composites with Cu_3_SbSe_4_ nanoinclusions to enhance energy filtering by creating interface potentials. Our results show that appropriate Pb-doping and Cu_3_SbSe_4_ nanoinclusions both increase PF owing to an increased thermopower and significantly reduce (approx. 2-fold) the thermal conductivity of β-Zn_4_Sb_3_. This results in a large ZT of up to 1.4 at 648K of the nanocomposite f(Cu_3_SbSe_4_)/β-Pb_0.02_Zn_3.98_Sb_3_ with f = 3 vol.% (where f is the volumetric percentage of Cu_3_SbSe_4_).

## Results and Discussion

### Thermoelectric properties

**Figure 1:**
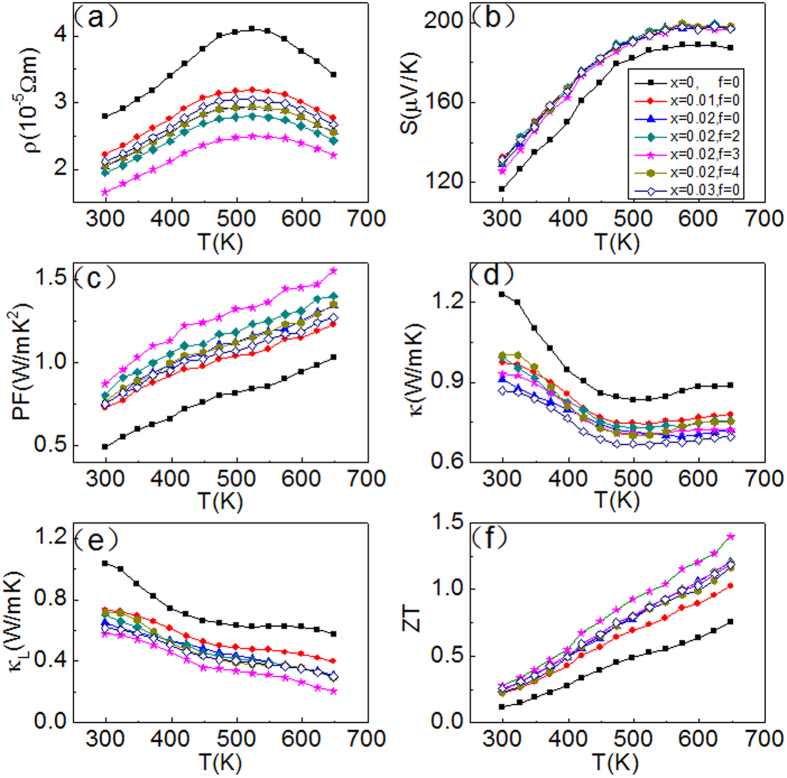
Temperature dependences of (**a**) electrical resistivity, (**b**) thermopower, (**c**) power factor, (**d**) total thermal conductivity, (**e**) lattice thermal conductivity, and (**f**) figure of merit ZT of β-Zn_4_Sb_3_, β-(Zn_1-x_Pb_x_)_4_Sb_3_ (x = 0.01, 0.02, and 0.03) and f(Cu_3_SbSe_4_)/β-Pb_0.02_Zn_3.98_Sb_3_ (f = 2, 3 and 4vol.%) composite samples.

The temperature dependences of the electrical resistivity ρ of β-Zn_4_Sb_3_, β-(Zn_1-x_Pb_x_)_4_Sb_3_ (x = 0.01, 0.02, and 0.03) and f(Cu_3_SbSe_4_)/β-Pb_0.02_Zn_3.98_Sb_3_ (f = 2, 3and 4 vol.%) nanocomposite samples are shown in Fig. 1(a). The resistivity of each sample increases with temperature and reaches to a maximum at around 550 K. Further increasing temperature leads to a decrease of ρ. This reduction might be mainly ascribed to the onset of mixed conduction or thermal excitation of minority carriers^18^. Moreover, it can be noticed that the resistivities of both Pb-doped β-Zn_4_Sb_3_ [β-(Zn_1-x_Pb_x_)_4_Sb_3_ (x = 0, 0.01, 0.02, and 0.03)] and the nanocomposite compounds [f(Cu_3_SbSe_4_)/β-Pb_0.02_Zn_3.98_Sb_3_ (f = 2, 3 and 4 vol.%)] are much smaller compared to pristine β-Zn_4_Sb_3_.

The carrier concentrations determined by Hall coefficient measurements are given in Table 1. It can be seen that with x increasing from 0 to 0.01 and 0.02, the hole concentration of β-(Zn_1-x_Pb_x_)_4_Sb_3_ increases from 12.1 to 13.6 and 16.9 × 10^19^ cm^−3^, respectively (see Table 1). With further increase of x to 0.03, the hole concentration slightly decreases to 15.2 × 10^19^ cm^−3^. As for the Cu_3_SbSe_4_ containing composites [f(Cu_3_SbSe_4_)/β-Pb_0.02_Zn_3.98_Sb_3_ (f = 2,3,4 vol.%)] (Table 1), the carrier concentration varies from 18.4 to 22.4 and 20.2 × 10^19^ cm^−3^ as the Cu_3_SbSe_4_ content increases from 2 to 3 and 4 vol.%, respectively. In addition, the mobility μ decreases moderately from 17.4 cm^2^/Vs to 15.1 cm^2^/Vs as f increases from 2 to 4 vol.%. These results indicate that the decrease of the resistivity with increasing inclusion content (see Fig.1 (a)) originates from the increased carrier concentration. Besides, the increase of the carrier concentration significantly exceeds the decrease of the mobility. The carrier concentration of f(Cu_3_SbSe_4_)/β-Pb_x_Zn_1-x_Sb_3_ for various values of x and f agrees with the resistivity trend in Fig.1 (a), indicating that the decrease of the resistivity with increasing doping and inclusion content originates from the changes of carrier concentration.

Figure 1(b) shows the temperature dependences of thermopower of β-Zn_4_Sb_3_, β-(Zn_1-x_Pb_x_)_4_Sb_3_ (x = 0.01, 0.02, and 0.03) and f(Cu_3_SbSe_4_)/β-Pb_0.02_Zn_3.98_Sb_3_ (f = 2, 3 and 4 vol.%) nanocomposite samples. Two points are particularly interesting: (1) Unlike the resistivity (Fig. 1(a)), the thermopower of all nanocomposite samples is nearly independent of the Pb and Cu_3_SbSe_4_ content; (2) From the observed increased carrier concentration (Fig.1(a)), a lower thermopower S of the Pb-doped samples and nanocomposite samples compared to pristine β-Zn_4_Sb_3_ would be expected. Instead, we find that the thermopower of these samples obviously increases in the whole temperature range implying an increase of N(E) or/and λ (energy filtering effect) according to Eq. 2 (see below).

Because of the enhanced thermopower and decreased resistivity, all β-(Zn_1-x_Pb_x_)_4_Sb_3_ (x = 0.01, 0.02, and 0.03) and f(Cu_3_SbSe_4_)/β-Pb_0.02_Zn_3.98_Sb_3_ (f = 2, 3 and 4 vol.%) samples have a higher power factor PF (=*S*^*2*^*/ρ*) than β-Zn_4_Sb_3_ in the whole temperature range (Fig. 1(c)). f(Cu_3_SbSe_4_)/β-Pb_0.02_Zn_3.98_Sb_3_ with f = 3 vol% shows the largest value with PF = 1.55 W/mK^2^ at 650 K, which is around 51% higher than that of pristine β-Zn_4_Sb_3_.

### Enhanced S by the resonant distortion of eDOS in Pb-doped β-(Zn_1-x_Pb_x_)_4_Sb_3_

From these results we assume that the anomalously enhanced thermopower [Fig. 1(b)] of the nanocomposite samples is due to resonant distortion of the eDOS and the EFM, respectively. Evidence of the resonant distortion of the eDOS will be provided by means of the Pb-doped samples. Based on the measured values of carrier concentration p and thermopower S, the effective mass m_d_* is calculated. In the single parabolic band model, m_d_* and S can be approximated by^33^,^34^:


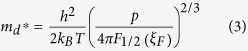



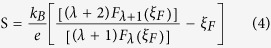


with the Fermi integral of order *i*


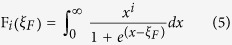


where h is the Planck constant, ξ_F_ is the reduced Fermi level F_f_/(k_B_T) and λ is the scattering parameter. As mentioned by Heremans *et al.*, the scattering parameter λ of doped systems without inclusions (or secondary phase) is dominated by the acoustic modes and can be zeroed^17^. Table 1 summarizes the resulting effective mass m_d_*/m_e_ (where m_e_ is the free electron mass). At 300 K, m_d_* of un-doped β-Zn_4_Sb_3_ is around 1.51 m_e_. The m_d_* of Pb-doped β-(Zn_1-x_Pb_x_)_4_Sb_3_ (x = 0.01, 0.02, and 0.03) reaches 1.91 m_e_, 2.14 m_e_, and 2.04 m_e_, respectively, which is 1.26, 1.42, and 1.35 times larger than that of the un-doped sample. The large effective mass indicates the strong resonant distortion of the eDOS around the Fermi level, since the eDOS is directly related to effective mass m_d_* (for instance, 
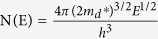
 for a free-electron gas)^35^.

**Figure 2:**
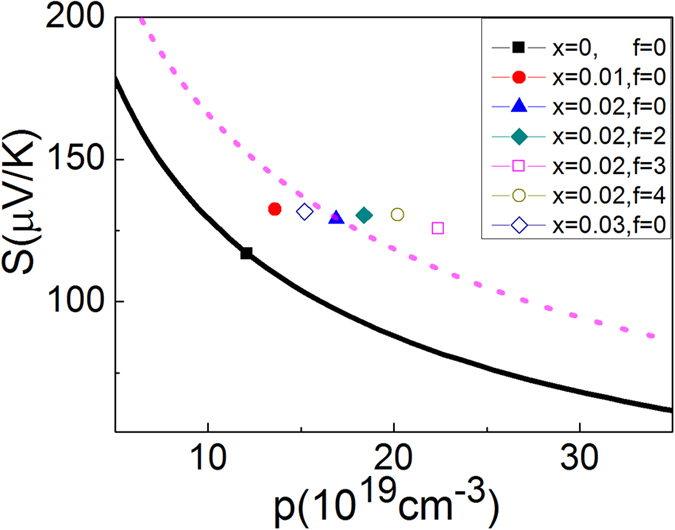
Variation of thermopower with carrier concentration of β-(Zn_1-x_Pb_x_)_4_Sb_3_ (0.01, 0.02, and 0.03) and f(Cu_3_SbSe_4_)/β-Pb_0.02_Zn_3.98_Sb_3_ (f = 2, 3 and 4 vol.%) at 300K. The black solid and pink dashed lines represent the carrier concentration dependence of thermopower (Pisarenko relation) of β-Zn_4_Sb_3_ using m_d_* = 1.51m_e_ and β-Pb_0.02_Zn_3.98_Sb_3_ using λ = 0, respectively.

Using formulae (3) and (4) and m_d_* = 1.51 m_e_ for the un-doped β-Zn_4_Sb_3_, we can plot the dependence of S on carrier concentration at 300K (black solid line in Fig. 2). Without resonant distortion of the eDOS Pb-doping, the thermopower S would be the same irrespective of the Pb-content and result in the same line. However, we find that S of β-(Zn_1-x_Pb_x_)_4_Sb_3_ with x = 0.01, 0.02, and 0.03 is ~23, 31 and 29 μV/K higher than the values of the black line, respectively (at 300K in Fig. 1(b)), indicating strong eDOS resonant distortion effects.

**Figure 3:**
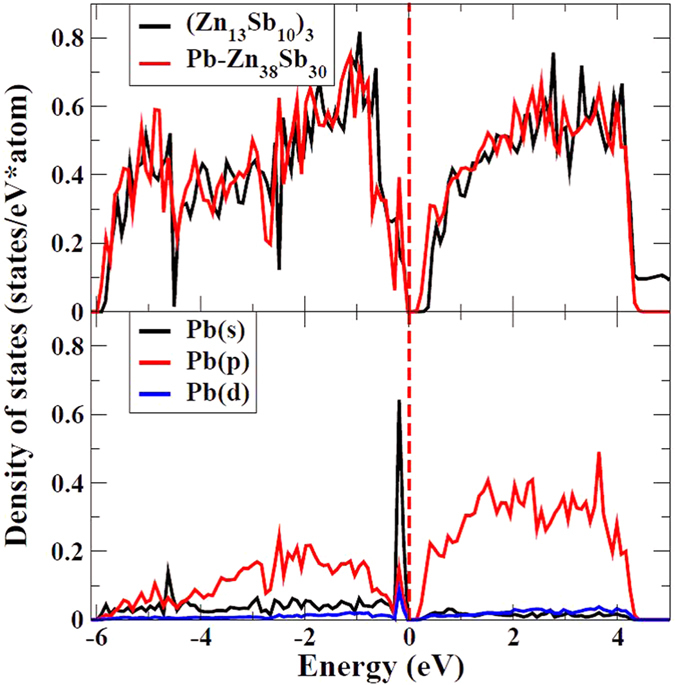
Top: Total electronic density of states (eDOS) of β-Zn_4_Sb_3_ (or Zn_13_Sb_10_) (black curve) and Pb-doped β-Zn_4_Sb_3_ (red curve). Bottom: The itemized electronic density of states (pDOS) of Pb of Pb-doped β-Zn_4_Sb_3_ compounds. The black, red and blue curves correspond to the pDOS of *s*, *p* and *d* states, respectively. The sharp peak around the Fermi level is dominated by the Pb s state.

The origin of the resonant eDOS distortion caused by Pb substitution is determined by first principle calculations of the energy bands of pristine β-Zn_4_Sb_3_ and Pb-doped β-Zn_4_Sb_3_ (Fig. 3). The calculated result indicates that Pb-doping induces a strong sharp resonant peak near the Fermi level, which is mainly dominated by the Pb s orbitals (bottom of Fig. 3). The Pb *p* orbitals contribute little to the peak, which is due to the transfer of the outmost *p* electrons from Pb to Sb. The sharp peak indicates a larger effective mass (m_d_*) and thermopower (S) of the Pb-doped system compared to pristine β-Zn_4_Sb_3_.

Furthermore, the resonant distortion of the eDOS of β-(Zn_1-x_Pb_x_)_4_Sb_3_ can also be quantified using the low-temperature heat capacity C_p_ of the samples. However, there are two temperature-dependent Zn_4_Sb_3_ modifications, i.e. β-Zn_4_Sb_3_ (T > 260 K) and α-Zn_4_Sb_3_ (T < 260 K), meaning that below ~260 K the β phase will transform to the α-phase. As a result, one can only measure low-temperature heat capacity C_p_ of α-Zn_4_Sb_3_. Nevertheless, previous work^36^ showed that the eDOS patterns of the two Zn_4_Sb_3_ modifications (β and α) are similar. Thus, it is appropriate to deduce the heat capacity (eDOS) of β-Zn_4_Sb_3_ from the α-Zn_4_Sb_3_ measurements. The temperature dependence of the low temperature (<4 K) heat capacity C_p_ of a solid is expressed by *C*_*p*_ = *γT* + *bT*^*3*^, where the term *bT*^*3*^ stands for the lattice contribution and *γT* for the charge carrier contribution with *γ* being related to N(E_f_) (eDOS at the Fermi level)^37^:


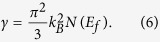


**Figure 4:**
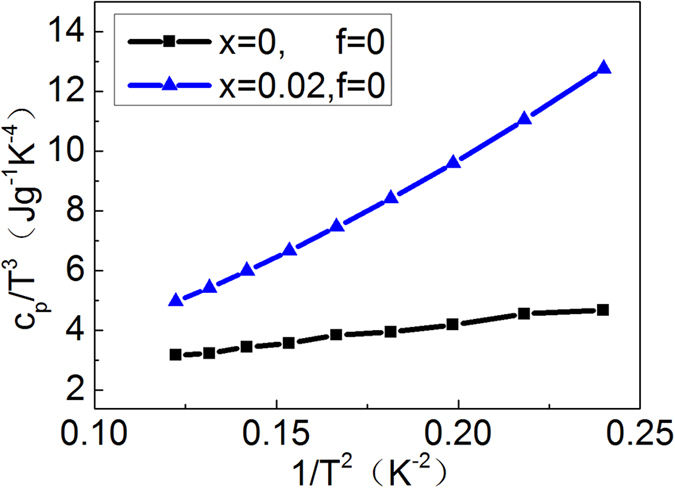
C_p_/T^3^ vs 1/T^2^ plots of Zn_4_Sb_3_ (α phase) and Pb_0.02_Zn_3.98_Sb_3_ (α phase).

Hence, the slope of a C_p_/T^3^ vs. 1/T^2^ plot gives *γ*, which is directly proportional to the eDOS at the Fermi level. Figure 4 shows the C_p_/T^3^ vs. 1/T^2^ plots of un-doped Zn_4_Sb_3_ (α phase) and a typical doped compound (Zn_1-x_Pb_x_)_4_Sb_3_ (*x* = 0.02) (α phase). The slope (*γ* ) of the plot of doped (Zn_1-x_Pb_x_)_4_Sb_3_ is substantially larger than that of un-doped Zn_4_Sb_3_. Linear fitting in the low temperature regime yields the ratio *γ*_dop_/*γ*_un-dop_ = N(E_f_)_dop_/N(E_f_)_0un-dop_ ~3.5 (see Fig. 4) revealing that Pb-doping indeed significantly increases the eDOS at Fermi level. This is in agreement with the first principle calculation result shown in Fig. 3.

### Energy filtering effect induced by Cu_3_SbSe_4_ nanoinclusions in f(Cu_3_SbSe_4_)/β-Pb_0.02_Zn_3.98_Sb_3_

We calculated the scattering parameters λ (Table 1) of the nanocomposites using the effective mass m_d_* = 2.14 m_e_ and the measured thermopwer S values. The results are comparable to the those obtained by Heremans *et al.* with Ag/PbTe nanocomposites^20^. Scattering parameters of the composite samples are throughout larger than that of β-Pb_0.02_Zn_3.98_Sb_3_ (see Table 1) suggesting a higher thermopower of the nanocomposite samples. The carrier concentration dependence of the thermopower S of β-Pb_0.02_Zn_3.98_Sb_3_ (λ = 0) at 300 K can be evaluated using formulae (3) and (4) yielding the pink dashed line in Fig. 2 (Pisarenko plot). Without energy filtering effect thermopower values of nanocomposites should lie on this pink line. Interestingly, the thermopower values of the f(Cu_3_SbSe_4_)/β-Pb_0.02_Zn_3.98_Sb_3_ (f = 2, 3 and 4 vol.%) nanocomposites at 300 K are above the line (~10, 15 and 12 μV/K, respectively) proofing the enhanced energy filtering effect.

**Figure 5:**
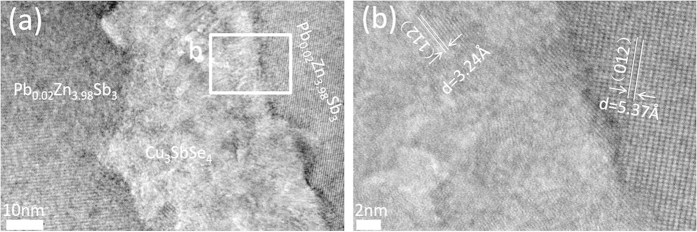
(**a**) Low-magnification bright-field image of f(Cu_3_SbSe_4_)/β-Pb_0.02_Zn_3.98_Sb_3_ with f = 3 vol.%. (**b**) lattice image of the rectangle area in (**a**)showing the β-Zn_4_Sb_3_ matrix (right), Cu_3_SbSe_4_ inclusions (left) and a typical phase boundary (between the matrix and the dispersed phase).

The above results indicate that the incorporation of nanophase Cu_3_SbSe_4_ in the Pb-doped β-Zn_4_Sb_3_ contributes to the large enhancement of S through the EFM. Microstructure analysis using high-resolution transmission electron microscopy (HRTEM) reveals the underlying mechanism. As shown in Fig.5(b), the Pb-doped β-Zn_4_Sb_3_ matrix and the dispersed Cu_3_SbSe_4_ particles are incoherently jointed at the phase boundary. Moreover, at room temperature the band gaps E_g_ of Cu_3_SbSe_4_ and Pb-doped β-Zn_4_Sb_3_ are 0.3–0.4 eV^31^,^38^ and 0.26 eV^39^, respectively, leading to a valence band offset and the formation of p-p-type heterojunction barriers at the phase boundary. It is reasonable to assume that these potential barriers act as scattering centers giving rise to the EFM^23^. Hence, the enhanced thermopower of f(Cu_3_SbSe_4_)/β-Pb_0.02_Zn_3.98_Sb_3_ results from the two effects: the resonant distortion of the eDOS in the Pb-doped β-Pb_0.02_Zn_3.98_Sb_3_ matrix and the energy filtering effect at the phase boundaries confirming that it is feasible to combine both effects in one system.

The temperature dependence of κ of β-Zn_4_Sb_3_, β-(Zn_1-x_Pb_x_)_4_Sb_3_ (x = 0.01, 0.02, and 0.03) and f(Cu_3_SbSe_4_)/β-Pb_0.02_Zn_3.98_Sb_3_ (f = 2, 3 and 4 vol.%) nanocomposite samples is shown in Fig.1(d). It can be seen that in the range of 300K to 500–550K, κ decreases with increasing temperature and then gradually increases as the temperature is further increased. κ includes the lattice thermal conductivity *κ*_*L*_ and the carrier contribution *κ*_*c*_: *κ* = *κ*_*L*_ + *κ*_*c*_. Thus, *κ*_*L*_ can be obtained by subtracting *κ*_*c*_ evaluated by the Wiedemann-Franz relation: *κ*_*c*_ = LT/*ρ*, where L is the Lorenz number. It is known that for heavily doped semiconductors, L is far below the Sommerfeld value L_0_ = 2.45 × 10^−8^ ΩWK^−2^, but depends on the reduced chemical potential ξ_F_, the band structure and the scattering process. In the single parabolic band model the Lorenz number is expressed as^33^:


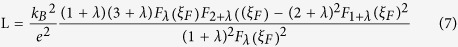


where ξ_F_ is obtained by fitting the measured S data using Eq.(4). The evaluated L(T) curve is plotted in Fig.S4 (Supplementary Information). Due to phonon scattering at both the doped sites and the boundaries, *κ*_*L*_ of all β-(Zn_1-x_Pb_x_)_4_Sb_3_ (x = 0.01, 0.02, and 0.03) and f(Cu_3_SbSe_4_)/β-Pb_0.02_Zn_3.98_Sb_3_ (f = 2, 3 and 4 vol.%) samples is lower than that of the pristine β-Zn_4_Sb_3_ (Fig. 1(e)). For instance, at 300K *κ*_*L*_ of the doped composite sample f(Cu_3_SbSe_4_)/β-Pb_0.02_Zn_3.98_Sb_3_ with f = 3 vol.% is only 0.58 W/Km, which is approx. 40% smaller than that of the β-Zn_4_Sb_3_ matrix.

Because of the simultaneous increase of PF and decrease of *κ*, the ZT values of all composite samples are enhanced compared to β-Zn_4_Sb_3_, as it is shown in Fig. 1(f). Specifically, 3 vol.% (Cu_3_SbSe_4_)/β-Pb_0.02_Zn_3.98_Sb_3_ reached ZT = 1.4 at 648 K, which is about twice as large as that of β-Zn_4_Sb_3_ studied here. This is the largest ZT value ever reported for a β-Zn_4_Sb_3_-based systems at 648 K^34^,^40^,^41^,^42^.

## Conclusions

We have demonstrated the enhancement of the thermoelectric properties as a result of two simultaneous effects: drastic reduction of the thermal conductivity and significant improvement of power factor. A figure of merit ZT = 1.4 at 648K could be achieved with *f*(Cu_3_SbSe_4_)/β-Pb_0.02_Zn_3.98_Sb_3_ with *f* = 3 vol.%, which is the largest ZT value ever reported in a β-Zn_4_Sb_3_-based systems at 648 K. The enhanced thermopower of β-Pb_0.02_Zn_3.98_Sb_3_-based composites with Cu_3_SbSe_4_ nanoinclusions results from the combination of resonant distortion of the eDOS in the Pb-doped matrix and intensified energy filtering at the heterojunction potential barriers. These findings provide a comprehensive way to design high-performance thermoelectric materials.

## Methods

β-(Zn_1-x_Pb_x_)_4_Sb_3_ (x = 0, 0.01, 0.02, and 0.03) samples were prepared from elemental Zn (99.9999%, powder), Sb (99.999%, powder) and Pb (99.9%, powder) in stoichiometric proportions. The elements were sealed in quartz tubes under vacuum (~10^−3^ Pa). The tubes were heated to 1023 K for 12 h and then quenched in cool water. The β-(Zn_1-x_Pb_x_)_4_Sb_3_ (x = 0, 0.01, 0.02, and 0.03) ingots were ground to powders by an agate mortar. To obtain Cu_3_SbSe_4_ powders, constituent elements CuCl (99%, powder), SbCl_3_(99%, powder) and Se(99%, powder) were put into a glass beaker containing ethylenediamine. Then the collected powder was filtered, washed and dried in a vacuum oven. The nanometer-sized Cu_3_SbSe_4_ and β-(Zn_1-x_Pb_x_)_4_Sb_3_ (x = 0, 0.01, 0.02, and 0.03) powders were mixed for 4 h in a volume ratio of 2:98, 3:97, 4:96 in a planetary mill. The disk-shaped bulk nanocomposites were obtained by hot-pressing the blended powders at 600MPa in vacuum at 650 K for 1 h.

X-ray diffraction (Philips-X PERT PRO) with Cu K_α_ radiation was used to check the phase constitutions. Scanning electron microscopy (SEM)(Hitachi S4800) equipped with an energy dispersive X-ray spectroscope (EDS) was used to analyze the microstructures of the composite samples. Moreover, microstructure investigations were also carried out using high-resolution transmission electron microscopy (HRTEM; JEOL JEM-2010) operating at a 200 kV accelerating voltage. Hall coefficients were measured by using a physical property measurement system (PPMS, Quantum Design). Low temperature heat capacity measurements were performed on the same instrument in the range of 2 K to 4 K. Electrical resistivity and thermopower were measured simultaneously by the standard four-probe method (ULVAC-RIKO: ZEM-3) in helium atmosphere from 300 K to 650 K. The thermal diffusivity α was measured with a NETZSCH LFA-457 instrument in the temperature range of 300 K to 650 K. The thermal conductivity 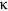 was calculated according to κ = DC_p_α, where C_p_ is the specific thermal capacity obtained by differential scanning calorimetry (DSC, perkin-Elmer) and D is the sample density measured by the Archimedes method.

DFT calculations were performed using the Vienna Ab Initio Simulation Package (VASP) with the projector augmented wave (PAW) scheme and the generalized gradient approximation of Perdew, Burke and Ernzerhof (GGA-PBE) for the electronic exchange-correlation functional. The energy cutoff for the plane wave expansion was 450 eV. The Brillouin zones were sampled by Monkhorst-Pack k-point meshes (3 × 3 × 2). Atomic positions and unit cell vectors were relaxed until all forces and components of the stress tensor were below 0.01 eV/Å and 0.2 kbar, respectively.

## Additional Information

**How to cite this article**: Zou, T. *et al.* Enhanced thermoelectric performance of β-Zn_4_Sb_3_ based nanocomposites through combined effects of density of states resonance and carrier energy filtering. *Sci. Rep.* **5**, 17803; doi: 10.1038/srep17803 (2015).

## Supplementary Material

Supplementary Information

## Figures and Tables

**Table 1:** Physical parameters of β-Zn_4_Sb_3_, β-(Zn_1-x_Pb_x_)_4_Sb_3_ (x = 0.01, 0.02, and 0.03) and f(Cu_3_SbSe_4_)/β-Pb_0.02_Zn_3.98_Sb_3_ (f = 2, 3 and 4 vol.%) at room temperature (300K).

f(vol.%)	a(A)^a^	c(A)^a^	p(10^19^cm^−3^)^b^	μ(cm^2^/Vs)^c^	m_d_*/m_e_^d^	λ^e^
x = 0, f = 0	12.207	12.420	12.1	18.6	1.51	0
x = 0.01, f = 0	12.220	12.423	13.6	20.7	1.91	0
x = 0.02, f = 0	12.224	12.431	16.9	18.1	2.14	0
x = 0.03, f = 0	12.227	12.420	15.2	19.4	2.04	0
x = 0.02, f = 2	12.220	12.423	18.4	17.4	2.14	0.18
x = 0.02, f = 3	12.220	12.423	22.4	16.8	2.14	0.30
x = 0.02, f = 4	12.220	12.423	20.2	15.1	2.14	0.28

^a^a and c are the lattice parameters.

^b^p is the hole concentration.

^c^μ is the Hall mobility.

^d^m_d_*/m_e_ is the ratio of the effective mass and the mass of the free electron.

^e^λ is the scattering parameter.
