# Supplementary material for: Enhanced thermoelectric performance of β-Zn4Sb3 based nanocomposites through combined effects of density of states resonance and carrier energy filtering
Source: Sci Rep. 2015 Dec 15;5:17803. doi: 10.1038/srep17803 (PMC4678945; doi:10.1038/srep17803)
Supplement: Supplementary Information [file srep17803-s1.pdf]

Supporting Information for

Enhanced thermoelectric performance of β-Zn_4Sb_3 based nanocomposites through combined effects of density of states resonance and carrier energy filtering

Tianhua Zou[1,2], Xiaoying Qin[1], Yongsheng Zhang[1,3], Xiaoguang Li[4,5], Zhi Zeng[1,3], Di Li[1], Jian Zhang[1], Hongxing Xin[1], Wenjie Xie[2], Anke Weidenkaff[2]

[1]*Key laboratory of Materials Physics, Institute of Solid State Physics, Chinese Academy of Sciences, 230031 Hefei, PR~China* [2]*Institute of Materials Science, University of Stuttgart, 70569 Stuttgart, Germany* [3]*University of Science and Technology of China, 230026 Hefei, PR~China* [4]*Hefei National Laboratory for Physical Sciences at Microscale, Department of Physics, University of Science and Technology of China, Hefei 230026, P.R.China* [5]*Collaborative Innovation Center of Advanced Microstructures, Nanjing University, Nanjing 210093, PR~China.*

Correspondence and requests for materials should be addressed to X. Q. (xyqin@issp.ac.cn) or Y. Z. (yshzhang@theory.issp.ac.cn)

1.Theoretical calculations

DFT calculations were performed using the Vienna Ab Initio Simulation Package (VASP) with the projector augmented wave (PAW) scheme[1] and the generalized gradient approximation of Perdew, Burke and Ernzerhof[2] (GGA-PBE) for the electronic exchange-correlation functional. The energy cutoff for the plane wave expansion was 450 eV. The Brillouin zones were sampled by Monkhorst-Pack[3] k-point meshes (3x3x2). Atomic positions and unit cell vectors were relaxed until all the forces and components of the stress tensor were below 0.01 eV/Å and 0.2 kbar, respectively.

From the experimental determinations[4], the β-Zn_4Sb_3 lattice constants are a=12.2282 Å and c=12.4067 Å with a R-3c space group. In the experimentally determined β-Zn_4Sb_3 unit cell, it contains 30 Sb atoms (the Wyckoff sites: 18e and 12c), but there are four inequilibrium Zn positions (Zn1, Zn2, Zn3 and Zn4 with the 36f Wyckoff site) with partial occupations: a deficiency of Zn1 site with ~90% occupancy and interstitial Zn2, Zn3 and Zn4 sites with ~5% occupancy to balance the stoichiometry. Thus the number of Zn in the unit cell is 39. From the experimental crystal structure analysis, the stoichiometry of β-Zn_4Sb_3 is actually $Zn_{39}Sb_{30}$ or $Zn_{13}Sb_{10}$. In order to perform DFT calculations on the β-Zn_4Sb_3 (or $Zn_{13}Sb_{10}$) compound, we should clearly know its atomic positions. To do so, we accept the structure construction strategy developed by Refs.[5,6] : Removing three Zn atoms from

the fully occupied Zn1 sites and inserting nine Zn atoms to Zn2 (three atoms), Zn3 (three atoms) and Zn4 (three atoms). This construction method makes sure that the distances of the three vacancies of Zn1 sites are as far as possible to have the lowest energy and the inserted interstitial sites form couple dimers to occupy the vacancies. The β-Zn_4Sb_3 (or $Zn_{13}Sb_{10}$) structure is illustrated in the left panel of Fig. S1. In the Pb substituted β-Zn_4Sb_3 calculations, we substituted one Pb for one Zn in various sites in the β-Zn_4Sb_3 unit cell, finding an energetic preference Pb position, substituting one of Zn2 positions (the right panel of Fig. S1).

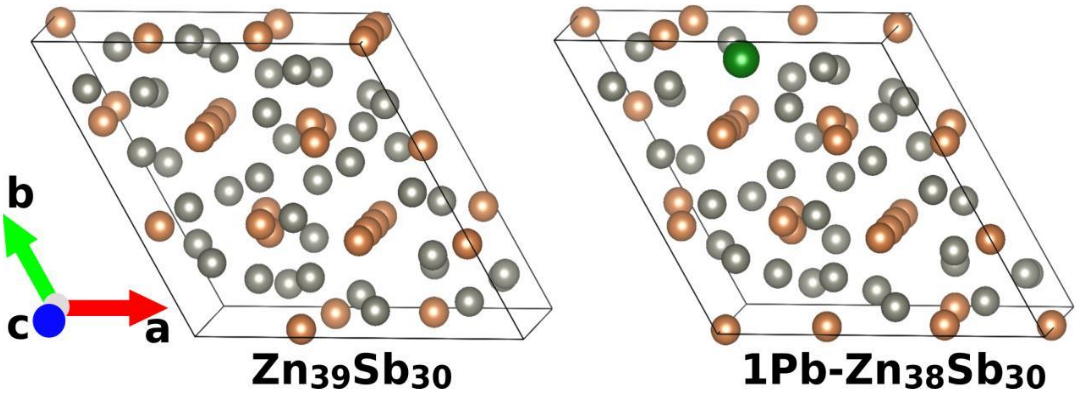

Fig. S1 Schematic of β-Zn_4Sb_3 (or $Zn_{39}Sb_{30}$) (the left panel) and the energetic favorite 1Pb-$Zn_{38}Sb_{30}$ (Pb substituting a Zn2 position, the right panel) crystal structures. Gray spheres, brown spheres and green spheres represent Zn, Sn and Pb atoms, respectively.

2.Microstructure characterization

Phase constitutions of Cu_3SbSe_4, β-Zn_4Sb_3, β-$(Zn_{1-x}Pb_x)_4Sb_3$ (x =0.01, 0.02, and 0.03) and f(Cu_3SbSe_4)/β-$Pb_{0.02}Zn_{3.98}Sb_3$ (f=2, 3and 4 vol.%) nanocomposite samples are analyzed by XRD, as shown in Fig. S2(A). From Fig. S2(A), all diffraction peaks of curve (a) and (b) can be perfectly indexed to the tetragonal Cu_3SbSe_4 structure (standard JCPDS number: 85-0003; space group I-42m) and to the rhombohedral β-Zn_4Sb_3 structure (standard JCPDS number: 89-1969; space group R-3c), respectively. This demonstrates that the pristine Cu_3SbSe_4 and β-Zn_4Sb_3 samples have been obtained in the study. The accurate lattice parameter measurements (Table 1 and Fig. S2(B)) reveal that the lattice constants of a and c of Pb doped samples are larger than those of undoped samples, which suggests that the substitutional compounds are

formed by substituting Pb for Zn; the larger atomic radius of Pb (Pb: ~1.80Å vs Zn: ~1.35 Å) leads to the expansion of host lattice. In the XRD patterns of f(Cu_3SbSe_4)/β-$Pb_{0.02}Zn_{3.98}Sb_3$ (f=2, 3and 4 vol.%) nanocomposites [(e), (f) and (g) in Fig. S2(A)], in addition to the XRD peaks of the matrix β-$Pb_{0.02}Zn_{3.98}Sb_3$, an additional small peak corresponding to Cu_3SbSe_4-(112) appears at 2θ=~27.4^o, which becomes increasing evidence with the increase of Cu_3SbSe_4 content. Thus, all peaks in the XRD patterns of f(Cu_3SbSe_4)/β-$Pb_{0.02}Zn_{3.98}Sb_3$ (f=2, 3 and 4 vol.%) composite samples are from β-Zn_4Sb_3 and Cu_3SbSe_4, no obvious impurity phases being observed in the composite samples.

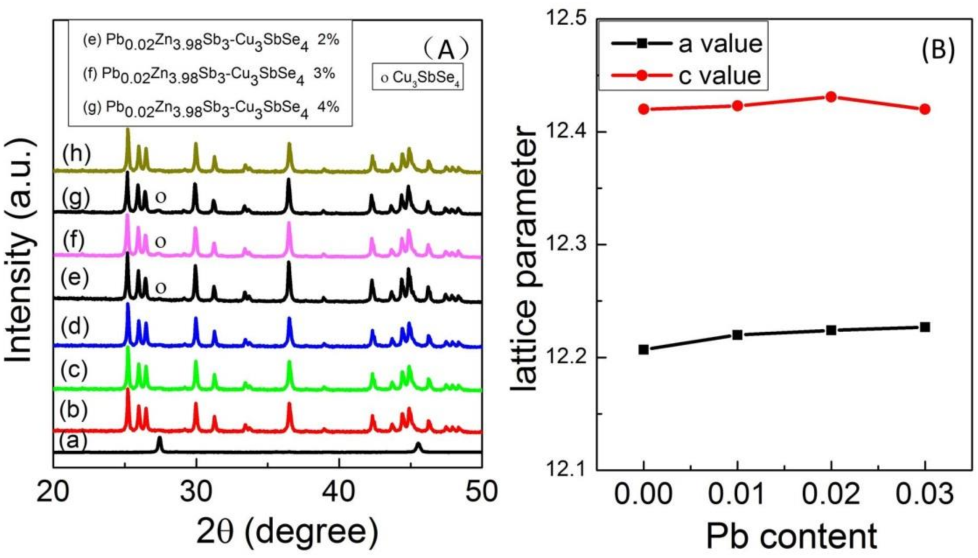

Fig. S2 (A)XRD patterns of (a) Cu_3SbSe_4, (b) β-Zn_4Sb_3 , (c)(g)(h)β-$(Zn_{1-x}Pb_x)_4Sb_3$ (x =0.01, 0.02, and 0.03) and (d-f)f(Cu_3SbSe_4)/ β-$Pb_{0.02}Zn_{3.98}Sb_3$ (f=2, 3and 4vol.%).(B)Pb content dependence of lattice parameters a and c for β-$(Zn_{1-x}Pb_x)_4Sb_3$ (x =0.01, 0.02, and 0.03).

Fracture surfaces of the sintered bulk composite sample f(Cu_3SbSe_4)/β-$Pb_{0.02}Zn_{3.98}Sb_3$ with f=3 vol.% are characterized by SEM and EDS, as shown in Fig. S3. The SEM observations clearly show that the sample consists of smooth areas (~5μm or larger in size) and granula regimes (with particles of submicrometers in size) (Fig. S3(a)),. Further analysis of the fractograph is made by EDS, as shown in Fig. S3 (b), (c) and (d), which shows that the chemical elements of spectrum 2 (explored in the smooth area) are just Zn and Sb, while spectrum 1

(explored in granula regime) and spectrum 3 (explored in granula regimes) contain Cu and Se, indicating that smooth areas (generating spectrum 2) is β-Zn_4Sb_3 and granula regimes (corresponding spectrum 1 and 3) contain Cu_3SbSe_4. These results further confirm that the mesoscopic Cu_3SbSe_4 particles are dispersed in the β-$Pb_{0.02}Zn_{3.98}Sb_3$ matrix.

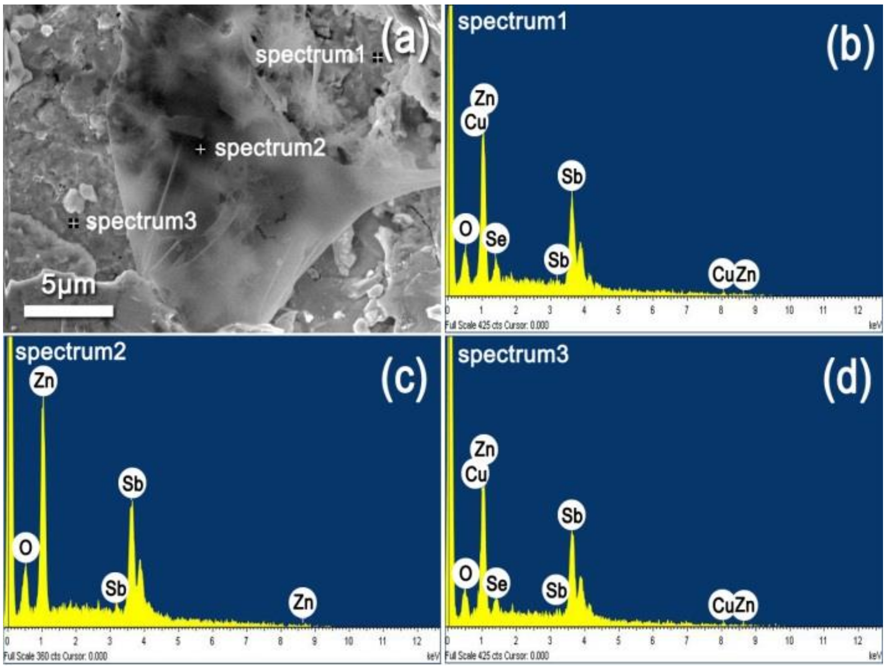

Fig. S3 SEM micrographs of (a) fracture surface of f(Cu_3SbSe_4)/β-$Pb_{0.02}Zn_{3.98}Sb_3$ with f=3 vol.% bulk composite sample, (b) EDS pattern detected in the granular area as indicated by spectrum 1 in (a), (c)EDS pattern detected in the smooth area as indicated by spectrum 2 in (a), and (d) EDS pattern detected in the granular area as indicated by spectrum 3 in (a).

3.Evaluation of Lorenz number

It is known that for heavily doped semiconductors, L is far below the Sommerfeld value L_0= $2.45\times10^{-8}\,\Omega\,WK^{-2}$, but dependent on reduced chemical potential ξ_F, the band structure and details of the scattering process. In single parabolic band model the Lorenz number is expressed as[7]:

$$L = \frac{k_B{}^2}{e^2}\frac{(1+\lambda)(3+\lambda)F_\lambda(\xi_F)F_{2+\lambda}((\xi_F)-(2+\lambda)^2F_{1+\lambda}(\xi_F)^2}{(1+\lambda)^2F_\lambda(\xi_F)^2}$$

Where ξ_F is obtained by fitting the measured S data using Eq.(4). The evaluated L(T) curve is plotted in Fig.S4. It is revealed that the Lorenz numbers are far below the L_0 marked by the top dotted line over the whole temperature range, owing to the

elastically scattered degenerate holes/electrons. And the values of L are very close to the non-degenerate limit marked by the bottom dotted line at high temperature.

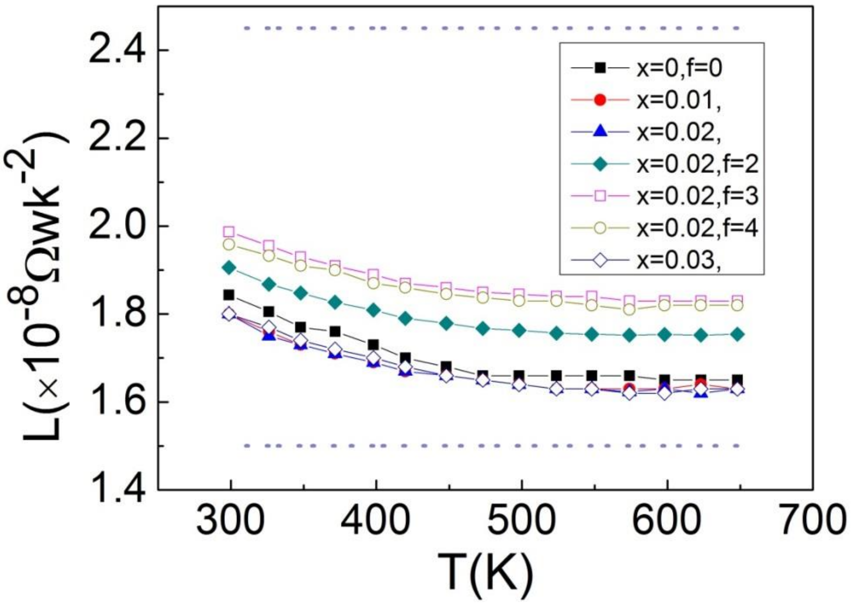

Fig. S4 Temperature dependence of evaluated Lorenz number L.

References:

1. Kresse, G. & Joubert, D. From ultrasoft pseudopotentials to the projector augmented-wave method. *Phys. Rev. B* **59,** 1758 (1999).
2. Perdew, J.P., Burke, K. & Ernzerhof, M. Generalized gradient approximation made simple. *Phys. Rev. Lett.* **77,** 3865 (1996).
3. Monkhorst, H.J. & Pack, J.D. Special points for Brillouin-zone integrations. *Phys. Rev. B* **13,** 5188-5192 (1976).
4. Snyder, G.J., Christensen, M., Nishibori, E., Caillat, T. & Iversen, B.B. Disordered zinc in Zn_4Sb_3 with phonon-glass and electron-crystal thermoelectric properties. *Nat. Mater.* **3,** 458-463 (2004).
5. Cargnoni, F. *et al.* Interstitial Zn atoms do the trick in thermoelectric zinc antimonide, Zn_4Sb_3: a combined maximum entropy method X-ray electron density and ab initio electronic structure study. *Chemistry* **10,** 3861-3870 (2004).
6. Qiu, A.N., Zhang, L.T. & Wu, J.S. Crystal structure, electronic structure, and thermoelectric properties of β-Zn_4Sb_3 from first principles. *Phys. Rev. B* **81,** 035203 (2010).
7. Xie, H. *et al.* Beneficial Contribution of Alloy Disorder to Electron and Phonon Transport in Half-Heusler Thermoelectric Materials. *Adv. Funct. Mater.* **23,** 5123-5130 (2013).
